# The British Medical Almanack for 1837

**Published:** 1837-01

**Authors:** 


ART. XI.—*The British Medical Almanack for* 1837; *with Supplement.* Edited by WILLIAM FARR.—Small 8vo. pp. 215. *London*, 1837.

IN our Journal of last year, we noticed, with just commendation, the volume of this work for 1836. A careful examination of that now before us authorises us to state that the present volume greatly excels its predecessors, excellent as they were, and justifies our recommendation of it to our readers in the strongest terms. The name of the editor is now made public for the first time; and assuredly the great industry, learning, and scientific knowledge displayed in this elaborate work are well calculated to enhance the reputation which he has already acquired by his statistical enquiries. Although the name of *Almanack* is appropriately given to this publication, yet, owing to the long degradation of this title, it is perhaps calculated to give to many an incorrect notion of Mr. Farr's work, which possesses claims to attention very different from those held out by the general class of slight and ephemeral productions so denominated. The great mass of intelligence of every kind contained in the first part of this little volume, all of it most interesting to the medical reader, is really extraordinary; while some of the statistical articles in the Supplement contain matter of the greatest importance. Our limits will only permit us to give a brief outline of its principal contents. The First Part, besides a Calendar for the year, contains a Medical Chronology, extending from the Arab schools to the discovery of the circulation by Harvey; an account of the Medical Colleges; lists of their officers or members, and of the licentiates admitted at Apothecaries' Hall, the College of Surgeons, and the University of Edinburgh, during the last year; the officers, regulations, and times of meeting, with a table, of the Medical and Scientific Societies; and a comprehensive view of all the Hospitals or Infirmaries and Dispensaries (with the exception of dispensaries in small towns,) in the kingdom. The Supplement contains the following articles:—1. A description of a new Hygrometer by Evaporation, (with a copper engraving,) invented by Dr. Mason. 2. On the History and future Prospects of the Provincial Medical and Surgical Association. 3. On the Statistics of Mortality in England, and the Statistics of Sickness, by T. R. Edmonds, B.A. 4. On Post-mortem Examinations, by the Rev. C. Oxendon. 5. The Weight and Stature of the Human Body at all ages, from M. Quetelet. 6. The Weight of the Brain at different ages, deduced from observations by Dr. Sims. 7. National Statistics: on the Incapacity of the Statistical Officers appointed by the English Government, the Errors of the Official Tables, &c. 8. Tables of Medical Weights all over Europe. 9. New Medicines, including an Epitome of Magendie's Formulary. 10. On the Mortality of the Officers in the English Army, and on the Deaths in different Months in the West Indies, New York, and the west coast of Africa. 11. On the Statistics of the British Hospitals; the best means of collecting their experience, and deducing results; a Table of the Patients admitted, resident, cured, and dying, in all the (sixty-five) British Hospitals and several Dispensaries; with Tables of the Mortality and Term of Treatment in several Hospitals. 12. On the Employment of Percussion and Auscultation in Diseases of the Chest. 13. Acts of Parliament, and miscellaneous articles.

